# Alterações respiratórias em crianças expostas à poeira de resíduos de mineração em Brumadinho, Minas Gerais, Brasil: Projeto Bruminha 

**DOI:** 10.1590/0102-311XPT131223

**Published:** 2024-02-26

**Authors:** Renan Duarte dos Santos Saraiva, Aline de Souza Espíndola Santos, Ana Paula Natividade de Oliveira, Maíra Lopes Mazoto, Volney de Magalhães Câmara, Carmen Ildes Fróes Rodrigues Asmus

**Affiliations:** 1 Instituto de Estudos em Saúde Coletiva, Universidade Federal do Rio de Janeiro, Rio de Janeiro, Brasil.; 2 Ministério da Saúde, Brasília, Brasil.; 3 Escola Nacional de Saúde Pública Sergio Arouca, Fundação Oswaldo Cruz, Rio de Janeiro, Brasil.; 4 Faculdade de Medicina, Universidade Federal do Rio de Janeiro, Rio de Janeiro, Brasil.

**Keywords:** Desastres, Poeira, Sinais e Sintomas Respiratórios, Doenças Respiratórias, Disasters, Dust, Respiratory Signs and Symptoms, Respiratory Tract Diseases, Desastres, Polvo, Signos y Síntomas Respiratorios, Enfermedades Respiratorias

## Abstract

Este estudo teve como objetivo investigar a ocorrência de afecções respiratórias em crianças expostas à poeira de resíduos de mineração após o desastre do rompimento da barragem em Brumadinho, Minas Gerais, Brasil. A população de estudo incluiu crianças com idades entre 0 e 6 anos, residentes em três comunidades expostas à resíduos de poeira de mineração (Córrego do Feijão, Parque da Cachoeira e Tejuco) e uma comunidade não exposta (Aranha). A coleta de dados ocorreu entre 19 e 30 de julho de 2021, por meio de questionários que abordavam informações sociodemográficas e um inquérito recordatório sobre sinais, sintomas e doenças respiratórias. Foram avaliadas 217 crianças, sendo 119 das comunidades expostas e 98 da comunidade não exposta. Os residentes nas comunidades expostas relataram aumento na frequência de faxina em suas residências (p = 0,04) e no tráfego de veículos (p = 0,03). Entre as crianças de 4 anos, foi observada uma maior frequência de afecções das vias aéreas superiores (p = 0,01) e inferiores (p = 0,01), bem como de alergia respiratória (p = 0,05). O grupo exposto apresentou 1,5 vez mais relatos de alergia respiratória (75%; p = 0,02) em comparação com o não exposto (50,5%). Crianças que viviam nas comunidades expostas à poeira de resíduos apresentaram três vezes mais chance (OR ajustada = 3.63; IC95%: 1,37; 9,57) de ocorrência de alergia respiratória em comparação com as não expostas. Dois anos e seis meses após a ocorrência do desastre ambiental, as crianças das comunidades afetadas pelos resíduos das atividades de mineração e remediação permaneciam expostas à poeira com efeitos tóxicos sobre a saúde respiratória.

## Abstract (en)

This study aimed to investigate the occurrence of respiratory diseases in children exposed to dust from mining waste after the Brumadinho dam disaster, Minas Gerais State, Brazil. The study population included children aged 0-6 years, living in three communities exposed to mining waste dust (Córrego do Feijão, Parque da Cachoeira, and Tejuco) and one unexposed community (Aranha). Data were collected from July 19 to 30, 2021, using questionnaires that addressed sociodemographic information and a recall survey on signs, symptoms, and respiratory diseases. A total of 217 children were evaluated, 119 living in the exposed communities and 98 in the non-exposed community. The residents in the exposed communities reported an increase in the frequency of home cleaning (p = 0.04) and in vehicular traffic (p = 0.03). Among children aged four, a higher frequency of upper (p = 0.01) and lower (p = 0.01) airway disorders, as well as respiratory allergy (p = 0.05) was observed. The exposed group had 1.5 times more reports of respiratory allergy (75%; p = 0.02) compared to the non-exposed group (50.5%). Children living in communities exposed to waste dust were three times more likely (adjusted OR = 3.63; 95%CI: 1.37; 9.57) to have respiratory allergies than those not exposed. Two years and six months after the environmental disaster occurred, children living in the communities affected by waste from mining and remediation activities remained exposed to dust with harmful effects on respiratory health.

## Abstract (es)

El objetivo de este estudio fue investigar la ocurrencia de enfermedades respiratorias en niños expuestos al polvo de residuos de la minería tras el desastre del colapso de la represa en Brumadinho, Minas Gerais, Brasil. La población de estudio incluyó niños que tenían entre 0 y 6 años, que viven en tres comunidades expuestas a residuos de polvo de la minería (Córrego do Feijão, Parque da Cachoeira y Tejuco) y una comunidad no expuesta (Aranha). Se recolectaron los datos entre el 19 y el 30 de julio de 2021, a través de cuestionarios que abordaban informaciones sociodemográficas y una encuesta recordatoria acerca de los señales, síntomas y enfermedades respiratorias. Se evaluaron 217 niños, de los cuales 119 viven en las comunidades expuestas y 98 viven en la comunidad no expuesta. Los residentes de las comunidades expuestas relataron un aumento en la frecuencia de limpieza de sus casas (p = 0,04) y en el tráfico de vehículos (p = 0,03). Entre los niños de 4 años, se observó una frecuencia más alta de enfermedades de las vías aéreas superiores (p = 0,01) e inferiores (p = 0,01), así como de alergia respiratoria (p = 0,05). El grupo expuesto presentó 1,5 veces más relatos de alergia respiratoria (el 75%; p = 0,02) en comparación con el grupo no expuesto (el 50,5%). Niños que vivían en las comunidades expuestas al polvo de residuos presentaron tres veces más probabilidad (OR ajustada = 3,63; IC95%: 1,37; 9,57) de ocurrencia de alergia respiratoria en comparación con los niños que no se expusieron. Dos años y seis meses tras el desastre ambiental, los niños que viven en las comunidades afectadas por los residuos de las actividades de minería y descontaminación permanecían expuestos al polvo con efectos tóxicos para la salud respiratoria.

## Introdução

A Organização Mundial da Saúde (OMS) alerta que 7 milhões de mortes prematuras são atribuídas à exposição aos poluentes atmosféricos, especialmente em países de baixa e média renda ^1^. Essa exposição pode causar efeitos no crescimento e na função pulmonar, além de infecções respiratórias em crianças ^1^. Diversos estudos apontam a pneumonia e a asma como as principais doenças relacionadas à poluição ambiental em crianças ^2^^,^^3^^,^^4^.

No Brasil, em 2022, as crianças menores de 5 anos de idade foram o grupo com mais internações hospitalares por doenças do aparelho respiratório (30,6%) em relação aos demais grupos etários. As causas mais frequentes de internação foram pneumonia (51,8%), bronquite e bronquiolite aguda (19,3%) e asma (8,7%). Ressalta-se que a COVID-19 não está classificada no capítulo X, que corresponde às doenças do aparelho respiratório ^5^.

A exposição a poluentes ambientais, especialmente a particulados ou aerodispersóides, durante a fase intrauterina, o nascimento e toda a infância pode oferecer danos à função pulmonar, considerando-se que o sistema respiratório ainda está em desenvolvimento e em maturação durante todo esse período ^6^. Diante dessa condição, deve-se considerar a exposição à poeira em geral e a resíduos de mineração, em particular, como uma preocupação para a saúde das crianças, uma vez que em sua composição existe uma diversidade de contaminantes químicos que são facilmente inalados ou ingeridos, sendo reconhecidamente agressores ao sistema respiratório ^7^.

Estudos como os de Branson ^8^, Glorennec et al. ^9^, Rasmussen et al. ^10^ e Shin et al. ^11^ identificaram os metais como compostos que podem ser constituintes da poeira. Adicionalmente, quando inaladas, as partículas em suspensão no ar podem ser transportadas até os alvéolos pulmonares, causando irritação do epitélio ^12^. Nesse sentido, os territórios com produção intensa e contínua de resíduos, devido às atividades industriais e de mineração, podem constituir uma importante fonte de exposição a poeiras para populações que vivem no entorno ^13^. As características fisiológicas e comportamentais das crianças possibilitam um maior contato com esses particulados que, por serem mais densos que o ar ambiente, depositam-se predominantemente na superfície do solo, podendo determinar maior absorção.

Em janeiro de 2019, ocorreu o rompimento da barragem B1 da mina do Córrego do Feijão, no Município de Brumadinho, Minas Gerais, Brasil. Tal desastre produziu uma avalanche de 12 milhões de m^3^ de rejeitos de minérios que atingiram uma vasta extensão territorial, causando 270 mortes e impactos ambientais, econômicos e sociais ^14^. Eventos como esse também produzem efeitos a longo prazo, com possíveis danos à população afetada, principalmente aos mais vulneráveis, como as crianças. É o caso da lama de rejeito depositada sobre as áreas das comunidades atingidas que, desde a ocorrência do desastre, secou e constituiu um novo solo superficial. É dessa camada de solo que se mobilizam os particulados mais finos (poeira) que possibilitam a exposição humana por meio da inalação ^15^^,^^16^. O material suspenso no ar e de granulometria mais fina pode alojar-se nos pulmões das crianças por meio da inalação e permanecer por longos períodos, resultando em uma maior absorção dos contaminantes ^15^.

O Projeto Bruminha é um estudo de coorte que avalia o impacto desse desastre sobre a saúde das crianças de 0 a 6 anos de idade residentes nas comunidades atingidas ao longo de 4 anos (2021 a 2024). Serão apresentados os resultados da avaliação da ocorrência de alterações respiratórias na população de estudo deste projeto, realizado no ano de 2021. Este artigo tem como objetivo investigar a ocorrência de afecções respiratórias em crianças expostas a resíduos de poeira de mineração nas comunidades afetadas.

## Método

### População de estudo

Estudo seccional com população composta por crianças na faixa etária de 0 a 6 anos de idade residentes nas comunidades de interesse, cujos responsáveis assinaram o Termo de Consentimento Livre e Esclarecido (TCLE).

### Área de estudo

**Figura 1:**
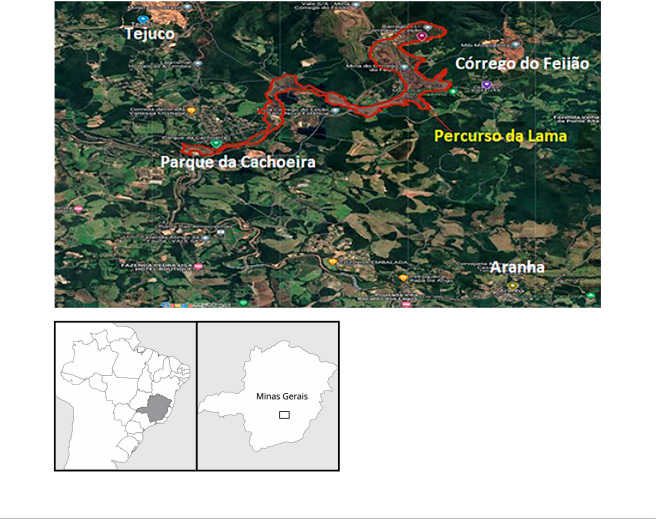
Localização das áreas de abrangência do estudo. Brumadinho, Minas Gerais, Brasil.

As localidades de interesse são as comunidades de Córrego do Feijão, Parque da Cachoeira, Tejuco e Aranha, do Município de Brumadinho. Foram consideradas populações expostas à poeira de mineração as residentes em Parque da Cachoeira, Córrego do Feijão e Tejuco e não expostas, as residentes em Aranha. As comunidades de Córrego do Feijão e Parque da Cachoeira estão localizadas em um raio de até 1,5km do trajeto percorrido pela lama e a comunidade de Tejuco está situada abaixo de uma área de mineração em atividade. A comunidade de Aranha está a uma distância de 10km da lama de rejeitos, a 11,6km de Córrego do Feijão, 11,3km de Parque da Cachoeira e 15,8km de Tejuco, sem área de mineração no seu entorno ^17^ (Figura 1).

### Fonte de informações

A coleta de dados foi realizada no período de 19 a 30 de julho de 2021. Foram aplicados questionários para levantamento de informações demográficas e de saúde das crianças e inquérito recordatório, respondido pelos pais e/ou responsáveis, considerando a ocorrência de sinais e sintomas para o período de 15 dias, e diagnóstico de doenças respiratórias pelo período de 12 meses.

(a) Questionário socioambiental: faixa etária, raça/cor (autorreferida), sexo, frequência de consumo de peixe, tipo de água utilizada para beber, destino do esgoto, escolaridade da mãe, renda *per capita*, aumento na frequência de faxinas para a retirada da poeira, tipo de pavimentação da rua, percepção de aumento da poeira, aumento do tráfego de veículos e hábito da criança de brincar com a terra.

(b) Formulário clínico: tosse, sibilo, dificuldade para respirar, congestão nasal/coriza, roncos/secreção, espirros recorrentes e otalgia (recordatório de 15 dias), pneumonia, asma/sibilância ou sibilo, bronquite, rinite/sinusite, alergia respiratória e otite (recordatório de 12 meses).

Esses instrumentos de coleta foram adaptados dos questionários geral e de eventos respiratórios do Projeto Infância e Poluentes Ambientais da Universidade Federal do Rio de Janeiro (Pipa/UFRJ). O questionário socioambiental foi aplicado por dois pesquisadores treinados e o formulário clínico por uma médica pediatra também pesquisadora do Projeto Bruminha (UFRJ). As entrevistas foram agendadas com auxílio dos profissionais da Secretaria Municipal de Saúde de Brumadinho e as perguntas foram direcionadas a mães e/ou responsáveis pelas crianças ^17^. É importante destacar que foi realizado um pré-teste dos questionários em uma subamostra da população de estudo e as falhas e imperfeições observadas foram corrigidas e incorporadas ao questionário final ^17^.

### Afecções respiratórias

As afecções respiratórias foram organizadas em: vias aéreas superiores (rinite/sinusite e otite), vias aéreas inferiores (pneumonia, asma/sibilância/sibilo e bronquite) e sinais e sintomas respiratórios (tosse, sibilo, dificuldade para respirar, congestão nasal/coriza, espirros recorrentes, roncos/secreções e otalgia). A alergia respiratória foi avaliada separadamente, pois não foi possível classificá-la quanto à sua localização nas vias aéreas superiores e inferiores. Essas informações não foram obtidas por meio de diagnóstico de profissionais de saúde ou por avaliação de prontuários, limitando essa classificação. Assim, adotou-se esse critério pressupondo-se que a alergia respiratória pode englobar e/ou ser confundida pelos responsáveis da criança como rinite/sinusite, bronquite ou asma.

### Análise dos dados

As variáveis categóricas foram descritas por frequências absolutas e relativas e foi realizado o teste qui-quadrado (testes exato de Fisher, de Yates e de Pearson) e mediana, intervalo interquartil (P25-P75) e teste de Mann-Whitney para variáveis numéricas. Associações foram avaliadas considerando um valor de p ≤ 0,05 como estatisticamente significativo.

Foi empregada a análise de regressão logística (*odds ratio* - OR bruta e ajustada) para estimar a associação entre as afecções respiratórias e a exposição a poeiras de resíduos de minério. Na construção do modelo ajustado, foram incorporadas as variáveis de confusão clássicas, como sexo, faixa etária e raça/cor da criança. Ainda, foi verificado por meio do teste qui-quadrado a ocorrência de diferença nas frequências entre os desfechos respiratórios pelas variáveis consumo de água e destino do esgoto, sendo observada diferença apenas para a variável destino do esgoto. Assim, o modelo ajustado final considerou as variáveis sexo, faixa etária, raça/cor e destino do esgoto. Para o processamento e análise dos dados, foi utilizado o software IBM SPSS Statistics, versão 20 (https://www.ibm.com/).

### Considerações éticas

Este estudo foi aprovado pelo Comitê de Ética em Pesquisa do Hospital Clementino Fraga Filho, vinculado à UFRJ (parecer nº 3.897.305).

## Resultados

Foram avaliadas 217 crianças, das quais 119 (54,8%) moram nas comunidades expostas a poeiras de resíduos de mineração (Tejuco, Parque da Cachoeira e Córrego do Feijão) e 98 (45,2%) residem na comunidade não exposta (Aranha). Eram do sexo masculino 52,5% (n = 114; p = 0,58), com uma média de idade de 43 meses (desvio padrão - DP: 23; p = 0,74). O grupo exposto apresentou predominância de crianças de raça não branca (p = 0,02) (Tabela 1), bem como maior frequência de faxinas para a retirada da poeira (n = 100; 89,3%; p = 0,04) e de aumento do tráfego de veículos (n = 105; 92,1%; p = 0,03) pós-desastre.

**Tabela 1:** Características da população residente nas áreas consideradas expostas e não expostas à poeira resultante do desastre do rompimento da barragem de mineração em Brumadinho, Minas Gerais, Brasil, 2021.

Características da população	Exposto	Não exposto	Valor de p	Total
n (%)	n (%)		n (%)
Faixa etária (meses)				
0-24	31 (26,1)	26 (26,5)	0,78 *	57 (26,3)
25-48	39 (32,7)	28 (28,6)		67 (30,9)
> 49	49 (41,2)	44 (45,8)		93 (42,8)
Subtotal	119 (100,0)	98 (100,0)		217 (100,0)
Raça/Cor				
Branca	34 (30,9)	44 (48,3)	0,02 **	78 (38,8)
Não branca	76 (69,1)	47 (51,7)		123 (61,2)
Subtotal	110 (100,0)	91 (100,0)		201 (100,0)
Sexo				
Masculino	60 (50,4)	54 (55,1)	0,58 **	114 (52,5)
Feminino	59 (49,6)	44 (44,9)		103 (47,5)
Subtotal	119 (100,0)	98 (100,0)		217 (100,0)
Consumo de peixe (dias/semana)				
Não consome	76 (71,7)	66 (75,0)	0,62 ***	142 (73,2)
1-3	28 (26,4)	19 (21,6)		47 (24,2)
4-7	2 (1,9)	3 (3,4)		5 (2,6)
Subtotal	106 (100,0)	88 (100,0)		194 (100,0)
Consumo de água				
Mineral	101 (89,4)	24 (25,3)	0,01 **	125 (60,1)
Outras fontes ^#^	12 (10,6)	71 (74,7)		83 (39,9)
Subtotal	113 (100,0)	95 (100,0)		208 (100,0)
Esgoto				
Fossa/Rede Geral	93 (86,1)	90 (96,8)		183 (91,1)
Outros ^##^	15 (13,9)	3 (3,2)	0,02 **	18 (8,9)
Subtotal	108 (100,0)	93 (100,0)		201 (100,0)
Escolaridade da mãe (anos de estudo)				
Nenhum	4 (3,5)	2 (2,1)	0,58 ***	6 (2,9)
1-9	31 (27,2)	32 (33,3)		63 (30,0)
> 9	79 (69,3)	62 (64,6)		141 (67,1)
Subtotal	114 (100,0)	96 (100,0)		210 (100,0)
Renda *per capita* [mediana/P25-P75]	500,0 (366,7; 747,5)	500,0 (292,8; 750,0)	0,72 ^###^	500,0 (322,5; 750,0)

Fonte: Projeto Bruminha (Universidade Federal do Rio de Janeiro).

* Qui-quadrado de Pearson;

** Qui-quadrado (teste de Yates; correção de continuidade);

*** Qui-quadrado (teste exato de Fisher);

^#^ Poço/Nascente/Cisterna;

^##^ Céu aberto/Rio/Lago;

^###^ Teste Mann-Whitiney.

A mediana da renda *per capita* foi de R$ 500,00 (P25-P75: 322,5; 750,0) (p = 0,72), com escolaridade materna acima de 9 anos em 67,1% (n = 141; p = 0,66) da população. O tipo de água utilizada para beber variou significativamente entre os grupos, assim como as condições de saneamento. O grupo exposto referiu maior consumo de água mineral (n = 101; 89,4%; p = 0,01) e destino inadequado do esgoto (n = 15; 13,9%; p = 0,02) (Tabela 1).

Ainda sobre a população geral, as crianças do sexo masculino apresentaram maiores queixas de afecções respiratórias para todos os desfechos avaliados, com 1,9 vez mais relatos de alergia respiratória, porém sem significância estatística. Em relação à faixa etária, nas crianças de 4 anos observou-se maior frequência de afecções respiratórias das vias aéreas superiores (p = 0,01), inferiores (p = 0,01) e alergia respiratória (p = 0,05) (Tabela 2).

**Tabela 2:** Ocorrência de afecções respiratórias segundo sexo e faixa etária na população de estudo em 2021.

Afecções respiratórias	Vias aéreas superiores *		Vias aéreas inferiores **		Sinais e sintomas respiratórios ***		Alergia respiratória	
	Sim	Não	Sim	Não	Sim	Não	Sim	Não
	n (%)	n (%)	n (%)	n (%)	n (%)	n (%)	n (%)	n (%)
Sexo								
Masculino	32 (53,3)	76 (51,4)	21 (58,3)	92 (51,1)	79 (54,9)	34 (47,2)	21 (65,6)	91 (50,0)
Feminino	28 (46,7)	72 (48,6)	15 (41,7)	88 (48,9)	65 (45,1)	38 (52,8)	11 (34,4)	91 (50,0)
Subotal	60 (100,0)	148 (100,0)	36 (100,0)	180 (100,0)	144 (100,0)	72 (100,0)	32 (100,0)	182 (100,0)
Valor de p	0,92 ^#^		0,54 ^#^		0,36 ^#^		0,15 ^#^	
Faixa etária (meses)								
0-24	6 (10,0)	50 (33,8)	3 (8,3)	54 (30,0)	35 (24,3)	22 (30,6)	1 (3,1)	55 (30,2)
25-48	13 (21,7)	51 (34,5)	7 (19,5)	59 (32,8)	45 (31,3)	21 (29,2)	12 (37,5)	53 (29,1)
> 49	41 (68,3)	47 (31,7)	26 (72,2)	67 (37,2)	64 (44,4)	29 (40,3)	19 (59,4)	74 (40,7)
Subotal	60 (100,0)	148 (100,0)	36 (100,0)	180 (100,0)	144 (100,0)	72 (100,0)	32 (100,0)	182 (100,0)
Valor de p	0,01 ^##^		0,01 ^##^		0,61 ^##^		0,05 ^##^	

Fonte: Projeto Bruminha (Universidade Federal do Rio de Janeiro).

* Rinite/Sinusite e otite;

** Pneumonia, asma/sibilância/sibilo e bronquite;

*** Tosse, sibilo, dificuldade para respirar, congestão nasal/coriza, espirros recorrentes, roncos/secreções e otalgia;

^#^ Qui-quadrado (teste de Yates; correção de continuidade);

^##^ Qui-quadrado de Pearson.

Em relação à ocorrência das afecções respiratórias, considerando os grupos exposto e não exposto à poeira de mineração, observou-se maior número de queixas referentes à ocorrência de afecções das vias aéreas superiores (n = 35; 58,3%; p = 0,62), das vias aéreas inferiores (n = 19; 52,8%; p = 0,95) e sinais e sintomas respiratórios (n = 78; 54,2%; p = 0,96) nas crianças do grupo exposto, com 1,5 vez mais relatos de alergia respiratória (75%; p = 0,02) em relação ao grupo não exposto (50,5%) (Tabela 3).

**Tabela 3:** Ocorrência de afecções respiratórias em crianças residentes nas áreas consideradas expostas e não expostas à poeira resultante do desastre do rompimento da barragem de mineração em Brumadinho, Minas Gerais, Brasil, 2021.

Afecções respiratórias	Sim	Não	Total	Valor de p
	n (%)	n (%)	n (%)	
Vias aéreas superiores *				0,62 **
Exposto	35 (58,3)	25 (41,7)	60 (100,0)	
Não exposto	79 (53,4)	69 (46,6)	148 (100,0)	
Vias aéreas inferiores ***				0,95 **
Exposto	19 (52,8)	17 (42,2)	36 (100,0)	
Não exposto	99 (55,0)	81 (45,0)	180 (100,0)	
Sinais e sintomas respiratórios ^#^				0,96 **
Exposto	78 (54,2)	66 (45,8)	144 (100,0)	
Não exposto	40 (55,6)	32 (44,4)	72 (100,0)	
Alergia respiratória				0,02 **
Exposto	24 (75,0)	8 (25,0)	32 (100,0)	
Não exposto	92 (50,5)	90 (49,5)	182 (100,0)	

Fonte: Projeto Bruminha (Universidade Federal do Rio de Janeiro).

* Rinite/Sinusite e otite;

** Qui-quadrado (teste de Yates; correção de continuidade);

^*^** Pneumonia, asma/sibilância/sibilo e bronquite;

^#^ Tosse, sibilo, dificuldade para respirar, congestão nasal/coriza, espirros recorrentes, roncos/secreções e otalgia.

Crianças vivendo nas comunidades expostas à poeira de mineração apresentam três vezes mais chances (OR ajustada = 3,63; IC95%: 1,37; 9,57) de ocorrência de alergia respiratória nos últimos 12 meses quando comparadas com as crianças da área não exposta (Tabela 4).

**Tabela 4:** Razão de chances para afecções respiratórias em crianças residentes em áreas expostas à poeira de resíduos de mineração comparadas às crianças em áreas não expostas à poeira. Brumadinho, Minas Gerais, Brasil, 2021.

Afecções respiratórias	OR bruta (IC95%)	OR ajustada * (IC95%)
Sinais e sintomas respiratórios **	0,94 (0,53; 1,67)	0,85 (0,44; 1,53)
Vias aéreas superiores ***	1,22 (0,67; 2,24)	1,26 (0,63; 2,55)
Vias aéreas inferiores ^#^	0,91 (0,45; 1,87)	0,85 (0,37; 1,97)
Alergia respiratória	2,93 (1,25; 6,87)	3,63 (1,37; 9,57)

Fonte: Projeto Bruminha (Universidade Federal do Rio de Janeiro).

* Ajustada por sexo, faixa etária, raça/cor e destino do esgoto;

** Tosse, sibilo, dificuldade para respirar, congestão nasal/coriza, espirros recorrentes, roncos/secreções e otalgia;

*** Rinite/Sinusite e otite;

^#^ Pneumonia, asma/sibilância/sibilo e bronquite.

## Discussão

Os resultados deste estudo indicam que a exposição à poeira de mineração pode ser um fator determinante na ocorrência de afecções respiratórias em crianças, com uma chance três vezes maior de desenvolvimento de processos alérgicos respiratórios naquelas que moram nas comunidades expostas aos resíduos, em comparação com as que não residem.

As atividades de remediação ainda em desenvolvimento e de extração de minério aumentam a poeira no ambiente intra e extradomiciliar. Os residentes da área exposta apresentam número significativamente maior de queixas quanto ao aumento da frequência de faxinas nas casas e do tráfego de veículos após a ocorrência do desastre. Crianças acima de 4 anos, faixa etária predominante na área exposta, geralmente têm maior acesso ao ambiente externo, o que resulta em uma maior exposição à poeira. Adicionalmente, os relatos de afecções respiratórias mais frequentes entre o sexo masculino podem estar associados a características culturais dos territórios, que determinam maior acesso dos meninos aos espaços externos e comunitários, por meio de jogos coletivos nas quadras de escolas e associações, praças e campos de futebol. Tais achados corroboram Esposito et al. ^6^, que ressaltam que as crianças, na maior parte do dia, estão em ambientes ao ar livre e praticam atividades físicas que estimulam o aumento da frequência respiratória, propiciando maior acúmulo de poluentes no trato respiratório. Soma-se a isso o fato de que o volume de ar inalado por minuto em relação ao peso corporal é em média o dobro do inalado por um adulto. Além disso, a árvore brônquica ainda está em estruturação, com uma menor quantidade de alvéolos pulmonares ^18^. Vale destacar que, durante o desenvolvimento intraútero de bebês, ocorrem diferenças entre meninos e meninas na formação dos órgãos, devido aos níveis hormonais característicos de cada sexo. Essas diferenças incluem a formação do sistema respiratório, em que os meninos apresentam maior resistência das paredes da faringe e traqueia, maior volume e tamanho pulmonar, bem como uma menor produção de surfactante em comparação com as meninas. Essas distinções se refletem na fisiologia respiratória, impactando em diferentes ritmos de frequência respiratória e na utilização de músculos respiratórios mais predominantemente em um sexo em relação ao outro ^19^.

Ainda do ponto de vista clínico, as diferenças anatômicas e fisiológicas da árvore respiratória e pulmonar podem ter impacto na predisposição a determinadas doenças do aparelho respiratório. Pressupõe-se que uma suscetibilidade diferente ao processo inflamatório induzida pelo sexo esteja presente na fase inicial da vida. Os hormônios femininos no início da puberdade e na menopausa podem apresentar relação com chiado no peito, falta de ar e tosse. Já entre os meninos, os hormônios masculinos e a formação anatômica do sistema respiratório promovem uma propensão maior à apneia obstrutiva do sono na vida adulta. Na população geral, a asma é mais prevalente em mulheres; porém, na infância, é mais prevalente entre os meninos ^19^, o que está em consonância com os achados deste estudo, que evidenciou maiores frequências de afecções respiratórias em crianças do sexo masculino.

Heinrich et al. ^20^ observaram, em um estudo transversal com 2.470 crianças entre 5 e 14 anos de idade, comparando expostas e não expostas à poluição industrial (mineração e fundição), que naquelas em áreas expostas à poluição industrial houve 50% de aumento na prevalência de alergias, eczema e bronquite, e cerca de duas vezes mais sintomas respiratórios, como chiado, falta de ar e tosse seca, em comparação às crianças residentes na área não exposta.

Ainda, Herrera et al. ^21^ realizaram um estudo em uma área de mineração de ouro e cobre a céu aberto no Chile e identificaram que esses processos de mineração estão associados ao aumento da exposição à poeira e, consequentemente, podem causar efeitos respiratórios em crianças. O estudo calculou o risco atribuível e estimou que se todas as 275 crianças participantes do estudo morassem a pelo menos um quartil de distância da mina, o risco de rinoconjuntivite alérgica reduziria em 4,7% e de rinoconjuntivite alérgica e asma combinados, em 4,2%. De forma geral, o estudo evidencia que aumentar a distância entre o local de residência das crianças e a fonte da geração dos resíduos reduz a prevalência de doenças respiratórias na comunidade em cerca de 4%.

Wichmann et al. ^22^ consideram que a exposição à poeira originária da poluição industrial apresenta efeitos mais nocivos sobre a árvore respiratória infantil do que àquela originária do tráfego de veículos. Em estudo realizado na cidade de La Plata, Argentina, os autores constataram que crianças expostas à poluição industrial apresentaram em torno de duas vezes mais chances de asma (OR = 2,76; IC95%: 1,96; 3,89), crise asmática (OR = 1,88; IC95%: 1,25; 1,83), sibilo (OR = 1,93; IC95%: 1,39; 2,67), dispneia (OR = 1,72; IC95%: 1,19; 2,48), tosse (OR = 1,76; IC95%: 1,29; 2,41) e rinite (OR = 1,87; IC95%: 1,12; 3,12), quando comparadas com crianças que viviam em outras áreas da cidade com poluição predominantemente relacionada ao trânsito.

Campos et al. ^23^, em investigação também realizada em Brumadinho com adultos, encontraram resultados similares aos observados neste artigo para a população infantil. A população que reside na área atingida diretamente pela lama do desastre (OR ajustada = 1,8; IC95%: 1,2; 2,5) e em áreas de mineração (OR ajustada = 1,6; IC95%: 1,1; 2,5) apresentou maiores chances de diagnóstico de asma. Ainda, foi verificada também maior chance de tosse seca (OR ajustada = 2,0; IC95%: 1,6; 2,6) e irritação nasal (OR ajustada = 2,3; IC95%: 1,9; 2,9).

As limitações deste estudo referem-se à coleta das informações apresentadas a partir dos relatos dos pais ou responsáveis pelas crianças, sem verificação em registros de atendimento de saúde. Os dados referentes às condições de saúde (sinais, sintomas e doenças respiratórias) não foram obtidos por meio de diagnóstico de profissionais de saúde ou por meio de avaliação de prontuários. Por outro lado, vale destacar que este estudo é aninhado a uma coorte, o Projeto Bruminha, que conta com financiamento e cronograma de execução aprovados para a realização de avaliações sobre a saúde respiratória das crianças ao longo de quatro anos. Nesse sentido, será possível acompanhar a evolução das condições de saúde da população de estudo ao longo desse período, possibilitando a observação da ocorrência de possíveis alterações.

Em suma, este estudo verificou que a exposição à poeira de resíduos de mineração está associada à maior chance de ocorrência de alergias respiratórias nas crianças com até 6 anos. Observou-se, também, que a exposição infantil a essas poeiras não ocorre exclusivamente durante o processo de extração de minério, mas também durante o processo de remediação e reparação dos impactos do desastre ocorrido em 2019. Espera-se que esses resultados possam contribuir para uma melhor estruturação dos serviços de assistência e vigilância em saúde nas comunidades que vivem próximas a áreas de mineração e são potencialmente expostas a poeiras de resíduos em todo o país.
