# ERRATUM: Alterações respiratórias em crianças expostas à poeira de resíduos de mineração em Brumadinho, Minas Gerais, Brasil:Projeto Bruminha.

**DOI:** 10.1590/0102-311XER131223

**Published:** 2025-06-27

Saraiva RDS, Santos ASE, Oliveira APN, Mazoto ML, Câmara VM, Asmus CIFR. Alterações respiratórias em crianças expostas à poeira de resíduos de mineração em Brumadinho, Minas Gerais, Brasil: Projeto Bruminha. Cad Saúde Pública 2024; 40(2):e00131223.

Onde se lê:

## Resumo:

Este estudo teve como objetivo investigar a ocorrência de afecções respiratórias em crianças expostas à poeira de resíduos de mineração após o desastre do rompimento da barragem em Brumadinho, Minas Gerais, Brasil. A população de estudo incluiu crianças com idades entre 0 e 6 anos, residentes em três comunidades expostas à resíduos de poeira de mineração (Córrego do Feijão, Parque da Cachoeira e Tejuco) e uma comunidade não exposta (Aranha). A coleta de dados ocorreu entre 19 e 30 de julho de 2021, por meio de questionários que abordavam informações sociodemográficas e um inquérito recordatório sobre sinais, sintomas e doenças respiratórias. Foram avaliadas 217 crianças, sendo 119 das comunidades expostas e 98 da comunidade não exposta. Os residentes nas comunidades expostas relataram aumento na frequência de faxina em suas residências (p = 0,04) e no tráfego de veículos (p = 0,03). Entre as crianças de 4 anos, foi observada uma maior frequência de afecções das vias aéreas superiores (p = 0,01) e inferiores (p = 0,01), bem como de alergia respiratória (p = 0,05). O grupo exposto apresentou 1,5 vez mais relatos de alergia respiratória (75%; p = 0,02) em comparação com o não exposto (50,5%). Crianças que viviam nas comunidades expostas à poeira de resíduos apresentaram três vezes mais chance (OR ajustada = 3.63; IC95%: 1,37; 9,57) de ocorrência de alergia respiratória em comparação com as não expostas. Dois anos e seis meses após a ocorrência do desastre ambiental, as crianças das comunidades afetadas pelos resíduos das atividades de mineração e remediação permaneciam expostas à poeira com efeitos tóxicos sobre a saúde respiratória.

## Palavras-chave:

Desastres

Poeira

Sinais e Sintomas Respiratórios

Doenças Respiratórias

## Abstract:

This study aimed to investigate the occurrence of respiratory diseases in children exposed to dust from mining waste after the Brumadinho dam disaster, Minas Gerais State, Brazil. The study population included children aged 0-6 years, living in three communities exposed to mining waste dust (Córrego do Feijão, Parque da Cachoeira, and Tejuco) and one unexposed community (Aranha). Data were collected from July 19 to 30, 2021, using questionnaires that addressed sociodemographic information and a recall survey on signs, symptoms, and respiratory diseases. A total of 217 children were evaluated, 119 living in the exposed communities and 98 in the non-exposed community. The residents in the exposed communities reported an increase in the frequency of home cleaning (p = 0.04) and in vehicular traffic (p = 0.03). Among children aged four, a higher frequency of upper (p = 0.01) and lower (p = 0.01) airway disorders, as well as respiratory allergy (p = 0.05) was observed. The exposed group had 1.5 times more reports of respiratory allergy (75%; p = 0.02) compared to the non-exposed group (50.5%). Children living in communities exposed to waste dust were three times more likely (adjusted OR = 3.63; 95%CI: 1.37; 9.57) to have respiratory allergies than those not exposed. Two years and six months after the environmental disaster occurred, children living in the communities affected by waste from mining and remediation activities remained exposed to dust with harmful effects on respiratory health.

## Keywords:

Disasters

Dust

Respiratory Signs and Symptoms

Respiratory Tract Diseases

## Resumen:

El objetivo de este estudio fue investigar la ocurrencia de enfermedades respiratorias en niños expuestos al polvo de residuos de la minería tras el desastre del colapso de la represa en Brumadinho, Minas Gerais, Brasil. La población de estudio incluyó niños que tenían entre 0 y 6 años, que viven en tres comunidades expuestas a residuos de polvo de la minería (Córrego do Feijão, Parque da Cachoeira y Tejuco) y una comunidad no expuesta (Aranha). Se recolectaron los datos entre el 19 y el 30 de julio de 2021, a través de cuestionarios que abordaban informaciones sociodemográficas y una encuesta recordatoria acerca de los señales, síntomas y enfermedades respiratorias. Se evaluaron 217 niños, de los cuales 119 viven en las comunidades expuestas y 98 viven en la comunidad no expuesta. Los residentes de las comunidades expuestas relataron un aumento en la frecuencia de limpieza de sus casas (p = 0,04) y en el tráfico de vehículos (p = 0,03). Entre los niños de 4 años, se observó una frecuencia más alta de enfermedades de las vías aéreas superiores (p = 0,01) e inferiores (p = 0,01), así como de alergia respiratoria (p = 0,05). El grupo expuesto presentó 1,5 veces más relatos de alergia respiratoria (el 75%; p = 0,02) en comparación con el grupo no expuesto (el 50,5%). Niños que vivían en las comunidades expuestas al polvo de residuos presentaron tres veces más probabilidad (OR ajustada = 3,63; IC95%: 1,37; 9,57) de ocurrencia de alergia respiratoria en comparación con los niños que no se expusieron. Dos años y seis meses tras el desastre ambiental, los niños que viven en las comunidades afectadas por los residuos de las actividades de minería y descontaminación permanecían expuestos al polvo con efectos tóxicos para la salud respiratoria.

## Palavras-claves:

Desastres

Polvo

Signos y Síntomas Respiratorios

Enfermedades Respiratorias

## Leia-se:

Resumo:

Este estudo teve como objetivo investigar a ocorrência de afecções respiratórias em crianças expostas à poeira de resíduos de mineração após o desastre do rompimento da barragem em Brumadinho, Minas Gerais, Brasil. A população de estudo incluiu crianças com idades entre 0 e 6 anos, residentes em três comunidades expostas à resíduos de poeira de mineração (Córrego do Feijão, Parque da Cachoeira e Tejuco) e uma comunidade não exposta (Aranha). A coleta de dados ocorreu entre 19 e 30 de julho de 2021, por meio de questionários que abordavam informações sociodemográficas e um inquérito recordatório sobre sinais, sintomas e doenças respiratórias. Foram avaliadas 217 crianças, sendo 119 das comunidades expostas e 98 da comunidade não exposta. Os residentes nas comunidades expostas relataram aumento na frequência de faxina em suas residências (p = 0,04) e no tráfego de veículos (p = 0,03). Entre as crianças de 4 anos, foi observada uma maior frequência de afecções das vias aéreas superiores (p = 0,01) e inferiores (p = 0,01), bem como de alergia respiratória (p = 0,05). O grupo exposto apresentou 3 vezes mais relatos de alergia respiratória (75%; p = 0,02) em comparação com o não exposto (25%). Crianças que viviam nas comunidades expostas à poeira de resíduos apresentaram três vezes mais chance (OR ajustada = 3.63; IC95%: 1,37; 9,57) de ocorrência de alergia respiratória em comparação com as não expostas. Dois anos e seis meses após a ocorrência do desastre ambiental, as crianças das comunidades afetadas pelos resíduos das atividades de mineração e remediação permaneciam expostas à poeira com efeitos tóxicos sobre a saúde respiratória.

## Palavras-chave:

Desastres

Poeira

Sinais e Sintomas Respiratórios

Doenças Respiratórias

## Abstract:

This study aimed to investigate the occurrence of respiratory diseases in children exposed to dust from mining waste after the Brumadinho dam disaster, Minas Gerais State, Brazil. The study population included children aged 0-6 years, living in three communities exposed to mining waste dust (Córrego do Feijão, Parque da Cachoeira, and Tejuco) and one unexposed community (Aranha). Data were collected from July 19 to 30, 2021, using questionnaires that addressed sociodemographic information and a recall survey on signs, symptoms, and respiratory diseases. A total of 217 children were evaluated, 119 living in the exposed communities and 98 in the non-exposed community. The residents in the exposed communities reported an increase in the frequency of home cleaning (p = 0.04) and in vehicular traffic (p = 0.03). Among children aged four, a higher frequency of upper (p = 0.01) and lower (p = 0.01) airway disorders, as well as respiratory allergy (p = 0.05) was observed. The exposed group had 3 times more reports of respiratory allergy (75%; p = 0.02) compared to the non-exposed group (25%). Children living in communities exposed to waste dust were three times more likely (adjusted OR = 3.63; 95%CI: 1.37; 9.57) to have respiratory allergies than those not exposed. Two years and six months after the environmental disaster occurred, children living in the communities affected by waste from mining and remediation activities remained exposed to dust with harmful effects on respiratory health.

## Keywords:

Disasters

Dust

Respiratory Signs and Symptoms

Respiratory Tract Diseases

## Resumen:

El objetivo de este estudio fue investigar la ocurrencia de enfermedades respiratorias en niños expuestos al polvo de residuos de la minería tras el desastre del colapso de la represa en Brumadinho, Minas Gerais, Brasil. La población de estudio incluyó niños que tenían entre 0 y 6 años, que viven en tres comunidades expuestas a residuos de polvo de la minería (Córrego do Feijão, Parque da Cachoeira y Tejuco) y una comunidad no expuesta (Aranha). Se recolectaron los datos entre el 19 y el 30 de julio de 2021, a través de cuestionarios que abordaban informaciones sociodemográficas y una encuesta recordatoria acerca de los señales, síntomas y enfermedades respiratorias. Se evaluaron 217 niños, de los cuales 119 viven en las comunidades expuestas y 98 viven en la comunidad no expuesta. Los residentes de las comunidades expuestas relataron un aumento en la frecuencia de limpieza de sus casas (p = 0,04) y en el tráfico de vehículos (p = 0,03). Entre los niños de 4 años, se observó una frecuencia más alta de enfermedades de las vías aéreas superiores (p = 0,01) e inferiores (p = 0,01), así como de alergia respiratoria (p = 0,05). El grupo expuesto presentó 3 veces más relatos de alergia respiratoria (el 75%; p = 0,02) en comparación con el grupo no expuesto (el 25%). Niños que vivían en las comunidades expuestas al polvo de residuos presentaron tres veces más probabilidad (OR ajustada = 3,63; IC95%: 1,37; 9,57) de ocurrencia de alergia respiratoria en comparación con los niños que no se expusieron. Dos años y seis meses tras el desastre ambiental, los niños que viven en las comunidades afectadas por los residuos de las actividades de minería y descontaminación permanecían expuestos al polvo con efectos tóxicos para la salud respiratoria.

## Palavras-claves:

Desastres

Polvo

Signos y Síntomas Respiratorios

Enfermedades Respiratorias

## Onde se lê:

Em relação à ocorrência das afecções respiratórias, considerando os grupos exposto e não exposto à poeira de mineração, observou-se maior número de queixas referentes à ocorrência de afecções das vias aéreas superiores (n = 35; 58,3%; p = 0,62), das vias aéreas inferiores (n = 19; 52,8%; p = 0,95) e sinais e sintomas respiratórios (n = 78; 54,2%; p = 0,96) nas crianças do grupo exposto, com 1,5 vez mais relatos de alergia respiratória (75%; p = 0,02) em relação ao grupo não exposto (50,5%) (Tabela 3).

Leia-se:

Em relação à ocorrência das afecções respiratórias, considerando os grupos exposto e não exposto à poeira de mineração, observou-se maior número de queixas referentes à ocorrência de afecções das vias aéreas superiores (n = 35; 58,3%; p = 0,62), das vias aéreas inferiores (n = 19; 52,8%; p = 0,95) e sinais e sintomas respiratórios (n = 78; 54,2%; p = 0,96) nas crianças do grupo exposto, com 3 vezes mais relatos de alergia respiratória (75%; p = 0,02) em relação ao grupo não exposto (25%) (Tabela 3).

## Onde se lê:

**Tabela 3:** Ocorrência de afecções respiratórias em crianças residentes nas áreas consideradas expostas e não expostas à poeira resultante do desastre do rompimento da barragem de mineração em Brumadinho, Minas Gerais, Brasil, 2021.

Afecções respiratórias	Sim	Não	Total	Valor de p
	n (%)	n (%)	n (%)	
Vias aéreas superiores *				0,62 **
Exposto	35 (58,3)	25 (41,7)	60 (100,0)	
Não exposto	79 (53,4)	69 (46,6)	148 (100,0)	
Vias aéreas inferiores ***				0,95 **
Exposto	19 (52,8)	17 (42,2)	36 (100,0)	
Não exposto	99 (55,0)	81 (45,0)	180 (100,0)	
Sinais e sintomas respiratórios ^#^				0,96 **
Exposto	78 (54,2)	66 (45,8)	144 (100,0)	
Não exposto	40 (55,6)	32 (44,4)	72 (100,0)	
Alergia respiratória				0,02 **
Exposto	24 (75,0)	8 (25,0)	32 (100,0)	
Não exposto	92 (50,5)	90 (49,5)	182 (100,0)	

Fonte: Projeto Bruminha (Universidade Federal do Rio de Janeiro).

* Rinite/Sinusite e otite;

** Qui-quadrado (teste de Yates; correção de continuidade);

^*^** Pneumonia, asma/sibilância/sibilo e bronquite;

^#^ Tosse, sibilo, dificuldade para respirar, congestão nasal/coriza, espirros recorrentes, roncos/secreções e otalgia.

Leia-se:

**Tabela 3:** Ocorrência de afecções respiratórias em crianças residentes nas áreas consideradas expostas e não expostas à poeira resultante do desastre do rompimento da barragem de mineração em Brumadinho, Minas Gerais, Brasil, 2021.

Afecções respiratórias	Sim	Não	Total	Valor de p
	n (%)	n (%)	n (%)	
Vias aéreas superiores *				0,62 **
Exposto	35 (58,3)	79 (53,4)	114 (54,8)	
Não exposto	25 (41,7)	69 (46,6)	94 (45,2)	
Vias aéreas inferiores ***				0,95 **
Exposto	19 (52,8)	99 (55,0)	118 (54,6)	
Não exposto	17 (47,2)	81 (45,0)	98 (45,4)	
Sinais e sintomas respiratórios ^#^				0,96 **
Exposto	78 (54,2)	40 (55,6)	118 (54,6)	
Não exposto	66 (45,8)	32 (44,4)	98 (45,4)	
Alergia respiratória				0,02 **
Exposto	24 (75,0)	92 (50,5)	116 (54,2)	
Não exposto	8 (25,0)	90 (49,5)	98 (45,8)	

Fonte: Projeto Bruminha (Universidade Federal do Rio de Janeiro).

* Rinite/Sinusite e otite;

** Qui-quadrado (teste de Yates; correção de continuidade);

^*^** Pneumonia, asma/sibilância/sibilo e bronquite;

^#^ Tosse, sibilo, dificuldade para respirar, congestão nasal/coriza, espirros recorrentes, roncos/secreções e otalgia.

